# Cardiovascular Magnetic Resonance Detects Myocardial Inflammation in Giant Cell Arteritis

**DOI:** 10.31138/mjr.32.4.363

**Published:** 2021-12-27

**Authors:** Sophie I. Mavrogeni, Athanasios Koutroumpas

**Affiliations:** 1 Onassis Cardiac Surgery Center, Athens, Greece,; 2 National and Kapodistrian University of Athens, Athens, Greece,; 3 Private practice, Volos, Greece

**Keywords:** GCA, CMR, tocilizumab, corticosteroids, sacubitril/valsartan

## Abstract

We describe a patient with GCA and cardiac involvement, presented as myocardial inflammation with reduced biventricular ejection fraction and diagnosed using cardiovascular magnetic resonance (CMR). She was treated successfully with sacubitril/valsartan in parallel with anti-rheumatic medication and the post-treatment CMR showed improvement of biventricular function and disappearance of myocardial inflammation.

## CASE REPORT

A 68-year-old woman was admitted to the hospital of Volos, internal medicine department, with fever (up to 39.5°C) despite pervious treatment with clarithromycin and, subsequently, amoxycillin/clavulanic acid. During hospitalisation, cardiac echocardiogram revealed reduced ejection fraction, and cardiac troponin was elevated. Thorough search for infectious causes of the fever was inconclusive, and the fever did not respond to several antibiotic regimens. At this time a temporal artery biopsy was performed that was positive for giant cell arteritis, and the patient was referred for rheumatological evaluation. Treatment with 32 mg methylprednisolone was instituted in June 2019. In parallel cardiac medication including b-blockers and ACE inhibitors was also started. After this treatment, the fever subsided, and laboratory markers of inflammation normalised. An echocardiogram performed in September 2019 showed LVEF=45–50%. Additionally, the first CMR in October 2019 showed reduced biventricular function, but myocardial inflammation was not identified, due to lack of collaboration with the patient. An echocardiogram in April 2020 showed LVEF=40.5%. After detailed instructions to the patient regarding how she should collaborate, a second CMR was performed in September 2020 and showed evidence of myocardial inflammation with reduced biventricular ejection fraction. Six months after the initial treatment, methotrexate was introduced to allow for faster tapering of corticosteroids. Tapering was successful, with no relapse in inflammatory markers, until September 2020, when the patient was receiving 3 mg methylprednisolone. Intriguingly, at this time inflammatory markers remained normal, but the third CMR evaluation showed myocardial inflammation and biventricular ejection fraction reduction. Methylprednisolone was re-escalated at 24 mg and tocilizumab was initiated (162 mg per week). In parallel treatment with Entresto (saccubitril+sartan) bid was started. A fourth CMR evaluation performed in January 2021 showed normalisation of biventricular ejection fraction and lack of myocardial inflammation. Finally, when we started the reduction of steroids, a 5^th^ CMR showed again evidence of inflammation, evidence of diffuse fibrosis and reduction of LVEF supporting that the intensive anti-rheumatic medication is crucial for maintaining the improvement in cardiac function. This fact obliged us to restart steroids, and the patient is waiting for re-evaluation in due course.

The evaluation of blood inflammatory indices (ESR and CRP) in parallel with the CMR study did not reveal evidence of inflammation. This is in agreement with our previous studies supporting that the blood inflammatory indices do not always mirror the presence of myocardial inflammation.^1^ Detailed CMR data and inflammatory indices are presented in **Table 1**.

**Table 1.** CMR parameters and inflammatory blood indices during the sequential CMR studies.

**CMR Parameters**	**1rst CMR (26-3-2019)**	**2^nd^ CMR (9-10-2019)**	**3rd CMR (28-9-2020)**	**4^th^ CMR (18-1-2021)**	**5^th^ CMR (STOP Steroids) (21-6-2021)**
**LVEDV (ml)**	145.4	146.5	163.9	197	243.6
**LVESV (ml)**	89.5	90.9	103.7	93	162.6
**LVEF (%)**	38.4	37.9	38.4	52.8	33.2
**RVEDV (ml)**	48.02	141.5	109.3	151.5	185
**RVESV (ml)**	33.1	77.7	84	90.3	103.6
**RVEF (%)**	30.9	45	23.1	41	43.9
**T2 RATIO (normal<2)**	1.02	1.4	3	1.2	1.9
**EGE (normal<4)**	2	5.9	6	2	4
**LGE (% LV)**	0	2 Subepicardial inferolateral wall	5 Subepicardial inferolateral wall	0	6 Subepicardial inferolateral wall
**T2 mapping (msec) (normal<50)**	34	44	58	42	56
**Native T1**	1112	1068	1288	1180	1291
**Mapping (msec) (normal<1200)**					
**ECV (%)(normal<29)**	23	25	23	24	30
**ESR (normal<20)**	30	15	31	15	10
**CRP (normal <5)**	0.7	2	0.3	0.9	0.8

GCA is a systemic vasculitis of large vessels, manifested mainly as temporal arteritis or large vessel vasculitis of the aorta and its branches. Myocarditis is a rare complication in GCA and all cases in the literature were diagnosed using positron emission tomography (PET).^2–7^ However, PET uses radiation, is extremely expensive and not widely available. Recently, CMR has been successfully used to diagnose myocarditis using both the classic Lake Louise criteria and the parametric imaging.^8^ To our knowledge, our paper is the second in the literature describing myocarditis during the course of GCA, diagnosed using CMR with evaluation before and after treatment. In the first paper, myocarditis was identified in 4/139 pts with Takayasu arteritis and GCA patients using CMR and aggressive immunosuppressive therapy led to remission of myocardial inflammation and improvement of LV function.^9^

Glucocorticoid therapy is essential and should be continued over a period of 1.5–2 years, with the cost of increasing adverse effects. Adjunctive methotrexate may reduce the cumulative glucocorticoid dosage by 20% to 44% and relapses by 36% to 54% in GCA.^10^ It has been recently published in NEJM that tocilizumab, received weekly or every other week, combined with a 26-week prednisone taper was superior to either 26-week or 52-week prednisone tapering plus placebo with regard to sustained glucocorticoid-free remission in patients with GCA.^11^ A significant reduction of cumulative glucocorticoid dose, prolonged relapse-free remission and reduced number of adverse events in the treatment groups have been demonstrated, supporting that tocilizumab can serve as effective glucocorticoid sparing agent.^11^ Furthermore, tocilizumab treatment may have contributed to the restoration of myocardial function, according to reports indicating improvement of myocardial function in RA patients following IL-6 inhibition.^12^

**Figure 1.**
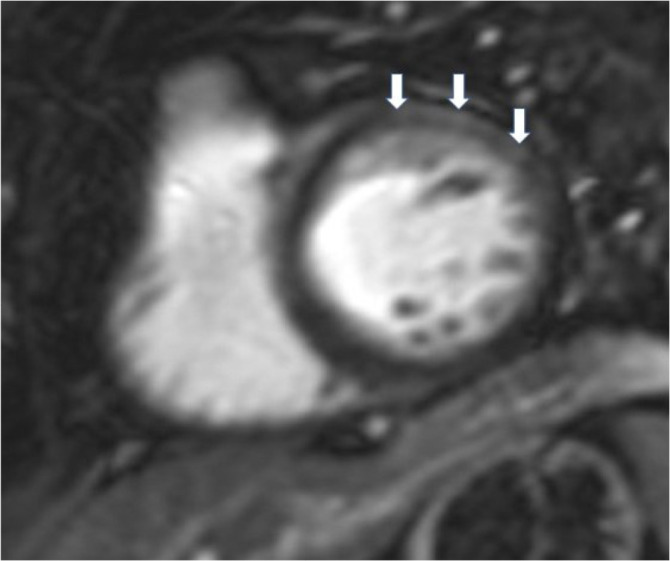
Inversion recovery short axis showing evidence of subepicardial late gadolinium enhancement (LGE) in the anterior wall of LV indicative of fibrotic process during myocardial inflammation (September 2020).

**Figure 2.**
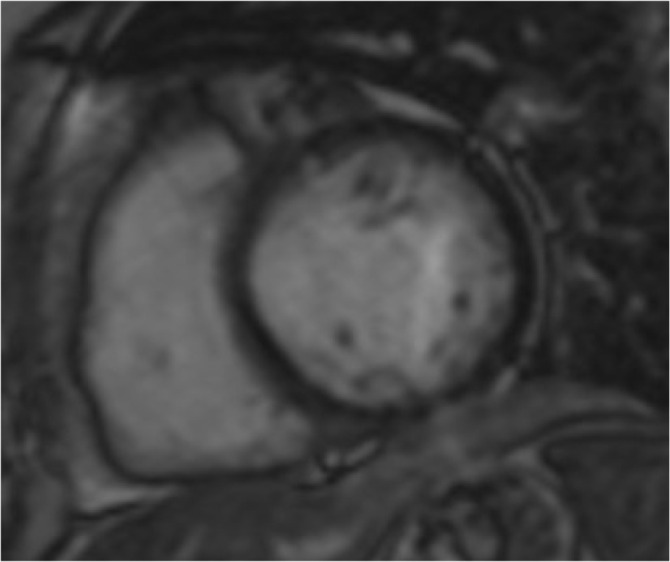
Inversion recovery short axis showing lack of LGE after anti-inflammatory and cardiac treatment (January 2021).

It is already known that long-term morbidity/mortality is improved in heart failure with reduced ejection fraction (HFrEF) using treatments targeting the renin-angiotensinaldosterone system (RAAS). Additionally, PARADIGM-HF, a large, phase III, randomized, controlled clinical trial proved that the enhancement of the activity of natriuretic peptides (NPs) can benefit patients with HFrEF. Sacubitril/valsartan is a first-in-class angiotensin receptor neprilysin inhibitor (ARNI) that simultaneously suppresses RAAS activation through blockade of angiotensin II type 1 receptors and enhances vasoactive peptides including NPs through inhibition of neprilysin, the enzyme responsible for their degradation. In PARADIGM-HF, patients with HFrEF treated with sacubitril/valsartan had 20% less risk for cardiovascular death or hospitalization for heart failure, 20% less risk for cardiovascular death, 21% less risk for first hospitalization for heart failure, and 16% less risk for death from any cause, compared with enalapril (all p < 0.001).^13^

The important findings of our case were:

1. The crucial role of CMR in the detection of myocardial inflammation, although the patient was asymptomatic and the inflammatory indices normal. In this context, CMR was the only non-invasive examination supporting the presence of myocardial inflammation although the clinical and laboratory evaluation was normal.
2. In comparison to echocardiography, CMR can provide reliable information about biventricular function in parallel with tissue characterisation for assessment of oedema, replacement, and diffuse fibrosis. This direct information of myocardial status can be used to individualise treatment protocols according to disease stage in each patient.
3. The significant improvement of ventricular function after treatment with sacubitril/valsartan in a patient where ACE inhibitors and b-blockers failed.

To our knowledge, our patient is the first in the literature that was diagnosed with GCA and treated successfully with sacubitril/valsartan in parallel with anti-rheumatic treatment. The significant improvement of biventricular function in our patient should encourage the use of sacubitril/valsartan in HFrEF patients, due to autoimmune rheumatic diseases (ARDs), in parallel with anti-rheumatic treatment.

## CONCLUSION

To conclude, CMR can reliably diagnose myocarditis in GCA using both the classic and the parametric criteria, even if the underlying disease is under control. The combination treatment with corticosteroids, tocilizumab and sacubitril/valsartan can dramatically improve the intractable HFrEF in GCA.
